# SUPRAPUBIC CATHETER INSERTION IN PEOPLE WITH MULTIPLE SCLEROSIS: LONG-TERM COMPLICATIONS AND SURVIVAL IN A RETROSPECTIVE COHORT STUDY

**DOI:** 10.2340/jrm.v58.45600

**Published:** 2026-06-04

**Authors:** Lisa GRANDIDGE, Amin Mohamed Abu BAKER, Steven KENNISH, Krishnan Padmakumari Sivaraman NAIR

**Affiliations:** 1 Sheffield Teaching Hospitals NHS Foundation Trust, Sheffield; 2 Department of Neurosciences, the University of Sheffield, UK

**Keywords:** catheterization, multiple sclerosis, neurogenic urinary bladder, neuropathic lower urinary tract dysfunction, suprapubic catheterization, survival analysis

## Abstract

**Objective:**

To evaluate complications and survival following radiologically guided suprapubic catheter insertion in people with multiple sclerosis.

**Design:**

Retrospective cohort study.

**Subjects/Patients:**

All patients with multiple sclerosis who underwent radiologically guided suprapubic catheter insertion at a tertiary regional multiple sclerosis centre in the United Kingdom between 2014 and 2020.

**Methods:**

Clinical and demographic data were obtained from medical records. Immediate complications occurring within 30 days and delayed complications occurring after 30 days were recorded. Kaplan–Meier and Cox regression analyses were performed to assess survival predictors.

**Results:**

Fifty-five patients underwent suprapubic catheter insertion. At the time of insertion, mean age was 62 years (standard deviation 11) and median duration of multiple sclerosis was 17.5 years (1^st^ quartile = 12.0; 3^rd^ quartile = 25.8). Immediate and delayed complication rates were 16% (9) and 27% (15), respectively. Five-year survival was 73%. Older age, higher disability scores, longer disease duration, and discharge to residential care were associated with poorer survival. Complications, sex, and mobility status were not associated with mortality.

**Conclusion:**

Radiologically guided suprapubic catheter insertion was associated with moderate complication rates and long-term survival in people with multiple sclerosis. These findings may allow better understanding of the potential complications of suprapubic catheterization in advanced multiple sclerosis.

## LAY ABSTRACT

Many people living with multiple sclerosis develop urinary bladder problems as the disease progresses. These problems can make it difficult to empty their urinary bladder properly and may lead to infections, leakage, and a reduced quality of life. When other treatments are no longer effective, a small tube called a suprapubic catheter can be placed directly into the urinary bladder through a small opening in the lower abdomen to help drain urine. In this study, we looked at people with multiple sclerosis who had this procedure and followed their health over several years. We found that the procedure was generally safe and that most people lived for many years after it was inserted. The results suggest that suprapubic catheters can be a useful long-term option for managing bladder problems in people with advanced multiple sclerosis, assisting physicians and patients in making informed treatment decisions.

Neurogenic lower urinary tract dysfunction (NLUTD) is common in people living with multiple sclerosis (MS) and is associated with significant physical burden and reduced health-related quality of life (1–4). NLUTD includes neurogenic detrusor overactivity (NDO), detrusor-sphincter dyssynergia, and impaired urinary bladder emptying, predisposing patients to urinary tract infections, urinary incontinence, and upper urinary tract deterioration (5).

Management strategies for NLUTD include behavioural modifications, pharmacological treatments, and intermittent urinary catheterization (IC) (1, 6, 7). However, as disability progresses, especially in those with impaired dexterity, cognition, or mobility, IC may no longer be feasible or effective, necessitating insertion of suprapubic catheters (SPCs), which are often placed percutaneously into the urinary bladder under radiological guidance (1, 8–10). Compared with in-dwelling urethral catheterization, SPCs are associated with fewer complications such as urethral trauma and catheter-associated urinary tract infections (CAUTIs) (11). They may facilitate improved hygiene, easier catheter care, and preservation of sexual function (1, 12). In patients with NLUTD or urinary continence issues related to poor mobility, limb weakness, or cognitive impairment, SPCs can improve quality of life (1, 12–14).

Despite their widespread use, data on long-term outcomes following SPC insertion in people with MS remain sparse (12, 15). Most existing studies include heterogeneous neurological populations, limiting their applicability to MS-specific care (16). Given the chronic, progressive nature of MS and the increasing use of SPCs as the disease advances, there is a need to better understand the long-term outcome of this intervention.

This retrospective study investigates the complications and survival following radiologically guided insertion of SPC in people with MS. Our aim is to generate data to inform clinical decision-making around the use of SPC in this population.

## METHODS

### Setting

This was a retrospective cohort study involving all patients with MS who underwent radiologically guided SPC insertion at a tertiary regional MS centre in Sheffield, UK. Patients were selected from institutional procedure records between August 2014 and March 2020. We excluded patients who did not have a diagnosis of MS.

Patients were followed from the date of SPC insertion until the occurrence of a defined event (death), or, for censored cases (those in whom the event was not observed during the follow-up period), until the last recorded follow-up in May 2022. No formal power calculation was performed prior to study initiation, as the aim was to include the entire eligible population within the defined study period, given the exploratory and descriptive nature of the study.

This study was reviewed and approved by the Clinical Effectiveness Unit of Sheffield Teaching Hospitals NHS Foundation Trust (STHCEU project number: 9932). The study did not involve any interventions or direct interaction with patients; hence consent was not required. The STROBE reporting checklist was used when writing this report (17).

### Data and outcome measures

Data were collected through a review of medical case notes and electronic hospital databases at Sheffield Teaching Hospitals NHS Foundation Trust. The following variables were collected: age, sex, MS subtype, duration of MS, mobility status, Expanded Disability Status Scale (EDSS) score at time of SPC insertion, procedure date, antimuscarinic medication use, discharge destination (home or residential care), and survival status (alive or deceased) after the procedure. We collected data on peri-procedural complications (occurring at the time of insertion), immediate complications (occurring within 30 days of SPC insertion), and delayed complications (occurring after 30 days of SPC insertion). Mortality data, including date and cause of death, were obtained from official death certificates issued by the General Register Office, UK. As data were obtained solely from hospital records, complications managed exclusively in primary care or by community nursing teams, such as catheter-associated urinary tract infections or catheter expulsions not requiring hospital attendance, may not have been fully captured. This represents a potential source of under-reporting and is acknowledged as a limitation of the study. All data were handled in accordance with institutional governance protocols and data protection regulations, including the UK General Data Protection Regulation (GDPR).

### Analysis

Statistical analysis was performed using IBM SPSS Statistics (version 28.0; IBM Corp, Armonk, NY, USA) (18) and JASP (version 0.18.3; https://jasp-stats.org/) (19). Descriptive statistics were used to summarize the cohort’s demographic and clinical characteristics. Missing data were handled using pairwise deletion, allowing for maximal use of available data while minimizing the loss of statistical power. This approach was the most appropriate given the exploratory nature of the study and the limited sample size (20).

Survival analysis was conducted using the Kaplan–Meier method to estimate time-to-event outcomes following SPC insertion, with log-rank (Mantel–Cox) tests applied to assess differences in survival distributions between groups. Kaplan–Meier survival curves with numbers at risk were generated using the survminer package (version 0.5.2) in R (version 4.5.0; R Foundation for Statistical Computing, Vienna, Austria). Cox proportional hazards regression was used to explore potential predictors of post-SPC mortality. The proportional hazards assumption was assessed using Schoenfeld residuals and visual inspection of log–log survival plots. Covariates explored in the regression model included age, sex, EDSS score, MS duration, presence of immediate and delayed complications, need for residential care, and mobility status. Hazard ratios (HRs) were reported as unadjusted HR (for univariable Cox regression model) and as adjusted HR (for multivariable Cox regression models after accounting for confounding variables).

Given the limited number of observed events (deaths) and the low number of events per variable (EPV), model complexity was restricted to a maximum of 2 covariates per regression model to mitigate the risk of overfitting. This approach aligns with established recommendations advocating for a minimum of 10 EPV to ensure model stability and interpretability (21).

## RESULTS

### Clinical and sociodemographic information

**Fig. 1:**
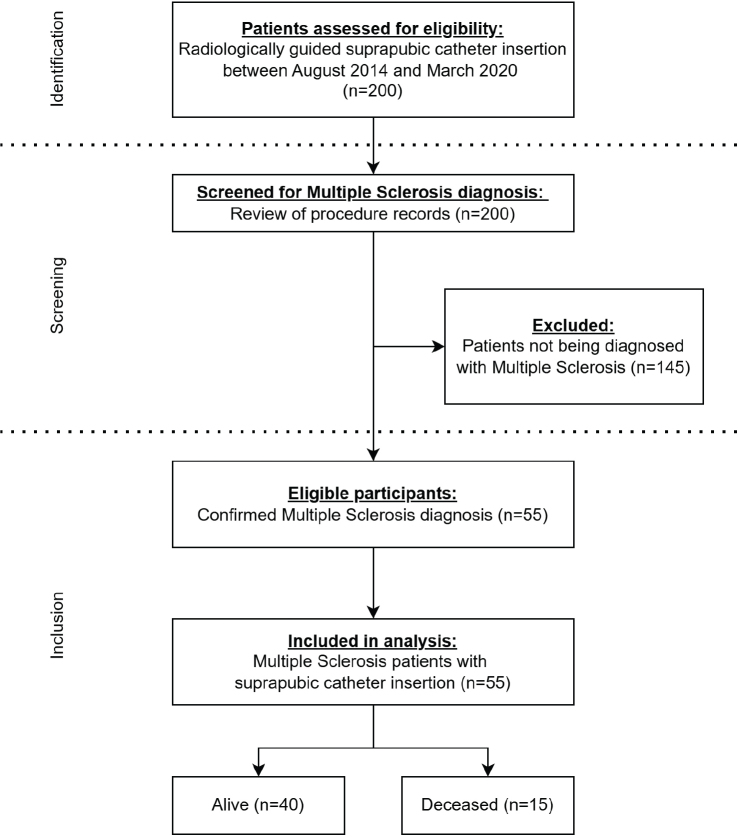
Study flow diagram according to STROBE criteria.

Of the 200 patients who underwent radiologically guided SPC insertion, 55 patients had a diagnosis of MS and were included in the study (Fig. 1).

Table I summarizes the demographic and clinical characteristics of patients with MS who underwent radiologically guided SPC insertion. Data were missing for mobility status in 2 patients. Little’s Missing Completely at Random (MCAR) test was not significant (*p* = 0.677), suggesting that missing values were likely MCAR. As missingness was consistent with MCAR, pairwise deletion was considered unlikely to introduce systematic bias. A complete-case sensitivity analysis (listwise deletion) was performed and yielded results consistent with the primary analysis. 60% (*n* = 33) of study patients were women. Seven (12.7%) had relapsing-remitting MS (RRMS), 7 (12.7%) had primary progressive MS (PPMS), and 38 (69.1%) had secondary progressive MS (SPMS). The subtype of MS was unclear from records in 3 patients (5.5%).

**Table I:** Descriptive statistics of radiologically inserted SPC MS patients

Parameters	MS patients (*n* = 55)
Age, years, mean (SD)	62.0 (11)
Duration of MS, years, median (Q1, Q3)	17.5 (12–26)
Sex: male, *n* (%)	22 (40)
Sex: female, *n* (%)	33 (60)
Age group, *n* (%)	
< 50 years)	8 (15)
50–59 years	12 (22)
60–69 years	23 (42)
70–79 years	9 (16)
≥ 80 years	3 (5)
Mobility status	
Walking, *n* (%)	15 (27)
Wheelchair, *n* (%)	37 (67)
Bedbound, *n* (%)	1 (2)
EDSS, median (Q1, Q3)	7.5 (7.0-8.0)
Immediate complications, *n* (%)	9 (16)
Delayed complications, *n* (%)	15 (27)
Discharge location	
Home, *n* (%)	49 (89)
Nursing home, *n* (%)	6 (11)

SPC: suprapubic catheter; SD: standard deviation; Q1: 1^st^ quartile; Q3: 3^rd^ quartile; EDSS: Expanded Disability Status Scale.

Median duration from MS diagnosis to SPC insertion was 17.5 years (1^st^ quartile [Q1]–3^rd^ quartile [Q3]: 12.0–25.8). The median EDSS score was 7.5 (Q1–Q3: 7.0-8.0); range 5.0–9.0. Some 42% (*n* = 23) of patients with MS were on antimuscarinic drugs prior to SPC insertion. The median follow-up duration for patients with MS following SPC insertion was 50 months (Q1–Q3: 36–70 months).

### Indications for SPC

The indications for SPC insertion were predominantly multifactorial for each patient (Table II). Inability or difficulty in performing IC was documented in 31 patients (56%), of whom 20 were reported to exhibit a general decline in motor function including reduced mobility, impaired hand function, and difficulty with transfers. Refractory lower urinary tract symptoms (LUTS) were noted in 29 patients (53%), encompassing symptoms such as urinary incontinence, urgency, frequency, incomplete urinary bladder emptying, nocturia, and overactive urinary bladder that were unresponsive to pharmacological therapy or botulinum toxin A detrusor injections. Four patients had an overactive urinary bladder, 3 of whom were later confirmed to have neurogenic detrusor overactivity (NDO) following urodynamic studies. Urodynamic data were not available for 1 patient; therefore, NDO could not be formally confirmed for them.

**Table II:** Indications for suprapubic catheter in multiple sclerosis patients (*n* = 55)

Indication	n* (%)
Inability/difficulty in performing intermittent catheterisation	31 (56)
Lower urinary tract symptoms (LUTS)	29 (53)
Urinary incontinence	12 (22)
Frequency	4 (7)
Incomplete urinary bladder emptying	4 (7)
Retention	3 (5)
Urgency	3 (5)
Overactive urinary bladder	1 (2)
Hesitancy	1 (2)
Nocturia	1 (2)
Problems with urethral catheter	19 (35)
Neurogenic detrusor overactivity (NDO)	3 (5)
Recurrent urinary tract infections	3 (5)
Cognitive impairment	2 (4)
Patient preference	2 (4)

* Indications were multifactorial; patients could have more than 1 indication. Percentages are calculated from the total cohort (*n* = 55) and therefore do not sum to 100%.

Prior to radiological SPC insertion, 37 patients (67%) were using a catheter: 19 (35%) had a prior indwelling urinary catheter (IUC) placed, and 17 (89%) of these underwent an elective transition to SPC. Sixteen (29%) patients were performing IC, and 3 (5%) had a pre-existing SPC that had fallen out, requiring replacement. One patient had previously used both an indwelling urethral catheter and an SPC. Eighteen patients (33%) had no prior catheter use, including 1 using a Conveen external sheath, predominantly due to urinary incontinence.

Baseline characteristics and outcomes were compared between patients with (*n* = 37) and without (*n* = 18) prior catheter use. The 2 groups were comparable in terms of mean age (60.7 vs 64.6 years, *p* = 0.213), median EDSS (7.5 vs 7.5, *p* = 0.234), median MS duration (18.0 vs 15.5 years, *p* = 0.818), mobility status (*p* = 0.092), delayed complications (*n* = 11 vs *n* = 4, *p* = 0.637), discharge to residential care (*n* = 4 vs *n* = 2, *p* = 0.973), and mortality (*n* = 8 vs *n* = 7, *p* = 0.177). Immediate complications occurred exclusively in patients with prior catheter use (*n* = 9 vs *n* = 0, *p* = 0.024).

### Immediate and delayed complications

Incidence of immediate complications (within 30 days of insertion of SPC) was 16.4% (*n* = 9 of 55) (Table III). The most frequent immediate complications in the whole cohort were infections (*n* = 6), 2 of which were of urinary source. One patient (1.8%) had SPC site infection; 3 (5.5%) had overnight admission due to pyrexia following SPC insertion, requiring intravenous antibiotics. One patient experienced a peri-procedural failed insertion attempt, successfully completed on the same visit. Occurrence of immediate complications did not affect survival (unadjusted HR = 1.00, *p* = 0.997; adjusted HR [age-adjusted] = 0.86, *p* = 0.821).

**Table III:** Immediate and delayed complications following suprapubic catheter (SPC) insertion in multiple sclerosis patients

Complications	*n* (%) *
Immediate complications (< 30 days)	9 (16.4)
Pyrexia/Infection (non-urinary)	4 (7.3)
Infection (urinary)	2 (3.6)
Catheter blockage	2 (3.6)
Deterioration in functional neurological symptoms	1 (1.8)
Delayed complications (> 30 days)	15 (27.3)
Infection (urinary)	6 (10.9)
Catheter blockage**	4 (7.3)
SPC fell out or accidentally removed	4 (7.3)
DVT and PE 3 months post-procedure	1 (1.8)

* Some patients experienced more than 1 complication. Percentages are calculated from the total cohort (*n* = 55).

** One patient with catheter blockage also experienced catheter bypassing.

DVT: deep vein thrombosis; PE: pulmonary embolism.

Fifteen (27.3%) cases of delayed complications were noted (see Table III). Occurrence of delayed complications did not predict survival in those with MS (unadjusted HR = 1.65, *p* = 0.361; adjusted HR (age-adjusted) = 2.38, *p* = 0.139). No patients experienced a worsening of MS symptoms in the first 2 weeks following SPC insertion. Two of the 4 SPC blockage events were caused by urinary bladder calculi. One patient had catheter bypassing secondary to SPC blockage.

No significant difference was observed in the median EDSS scores between patients who experienced immediate complications and those who did not (Yes: EDSS 7.0; No: EDSS 7.5; Mann–Whitney *U* = 262.5, *p* = 0.119). A similar finding was noted for delayed complications, with identical median EDSS scores in both groups (Yes: EDSS 7.5; No: EDSS 7.5; Mann–Whitney *U* = 240.0, *p* = 0.424).

### Survival

During follow-up, 27% (*n* = 15) of patients died after SPC insertion. The Kaplan–Meier mean survival time for the cohort was 5.7 years (SE 0.29, 95% CI: 5.1–6.3). The overall median survival time could not be estimated owing to the high proportion of censored data, with many patients surviving beyond the study period. Among the patients who died, the median time from SPC insertion to death was 870 days (Q1–Q3: 792–1,385 days).

**Fig. 2:**
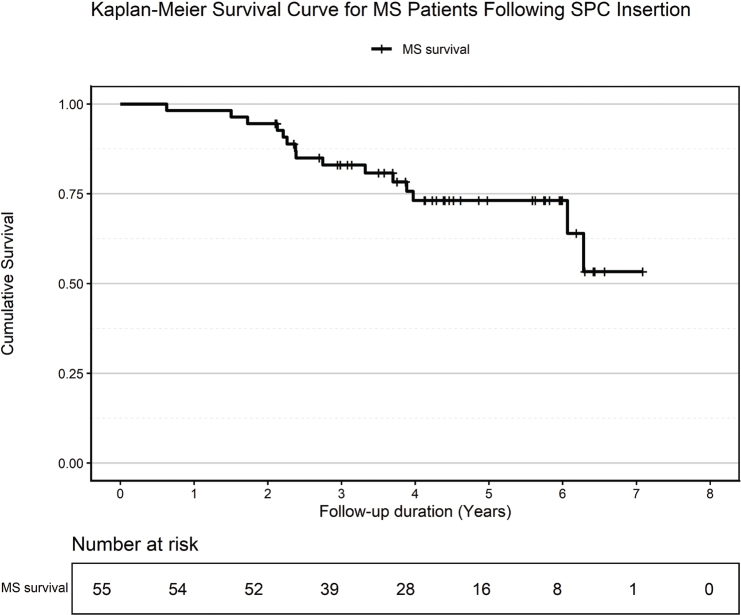
Survival curve for multiple sclerosis (MS) patients following suprapubic catheter (SPC) insertion.

Only 1 patient died within a year of SPC insertion (1-year survival rate of 98%). The 2-year survival rate was 92.7% (SE 0.035) and the 5-year survival rate was 73.1% (SE 0.062). Fig. 2 shows the Kaplan–Meier survival curve for the MS-SPC cohort.

**Fig. 3:**
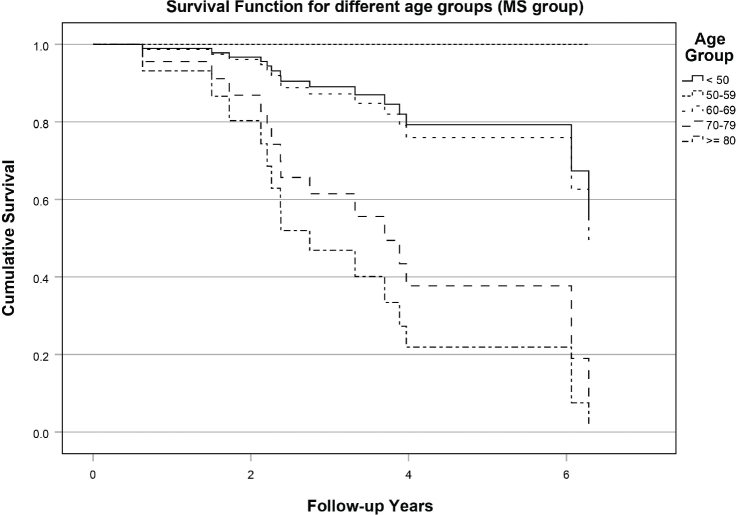
Kaplan–Meier cumulative survival functions as per different age groups for multiple sclerosis (MS) patients.

Age (ungrouped, continuous covariate) was a significant predictor of survival, with an unadjusted HR of 1.11 per year increase (*p* < 0.001). This association remained significant after adjusting for duration of MS diagnosis (adjusted HR = 1.12, *p* = 0.004). A clear distinction was also noted between the survival of patients in different age groups (log-rank [Mantel–Cox]: χ² = 14.868, *p* = 0.005) (Fig. 3). Those in the age groups of 70 and above had higher predicted hazard ratios for mortality than the other age groups in the cohort (70–79: unadjusted HR = 4.20, *p* = 0.105; adjusted HR = 2.09, *p* = 0.427; > 80: unadjusted HR = 6.54, *p* = 0.078; adjusted HR = 4.45, *p* = 0.240) though these were not statistically significant.

Due to the small sample size and limited events in certain EDSS scores, EDSS scores were grouped into 3 clinically relevant categories: EDSS 5.0–6.5 (*n* = 12, 22%), EDSS 7.0–8.0 (*n* = 38, 69%), and EDSS 8.5–9.5 (*n* = 5, 9%). These groupings were determined post-hoc based on clinical relevance and were not pre-specified. Kaplan–Meier survival analysis demonstrated a significant difference in survival distributions across EDSS groups (log-rank: χ² = 7.143, *p* = 0.028). Mean survival time decreased with increasing disability: 6.23 years (95% CI: 5.14–7.32) for EDSS 5.0–6.5; 5.65 years (95% CI: 5.13–6.17) for EDSS 7.0–8.0; and 3.47 years (95% CI: 1.78–5.16) for EDSS 8.5–9.5. Median survival time could not be estimated in the first 2 EDSS groups due to a high proportion of censoring, but was estimated at 2.26 years (95% CI: 0.33–4.19) in the 8.5–9.5 EDSS category. In the Cox proportional hazards model, higher EDSS scores were significantly associated with increased risk of mortality following SPC insertion (unadjusted HR = 2.57, *p* = 0.029; adjusted HR [age-adjusted] = 3.17, *p* = 0.032).

Duration since MS diagnosis showed a small but significant impact on survival post-SPC insertion (unadjusted HR = 1.01, *p* = 0.030; adjusted HR [age-group adjusted] = 1.01, *p* = 0.045). However, the adjusted HR was not significant when corrected for other confounding variables: age (ungrouped) (adjusted HR = 1.01, *p* = 0.053); EDSS (adjusted HR = 1.00, *p* = 0.188); and discharge to residential care (adjusted HR = 1.00, *p* = 0.075). Sex did not impact survival in the MS-SPC patients (unadjusted HR = 2.17, *p* = 0.147; adjusted HR [age-adjusted] = 2.61, *p* = 0.072).

**Fig. 4:**
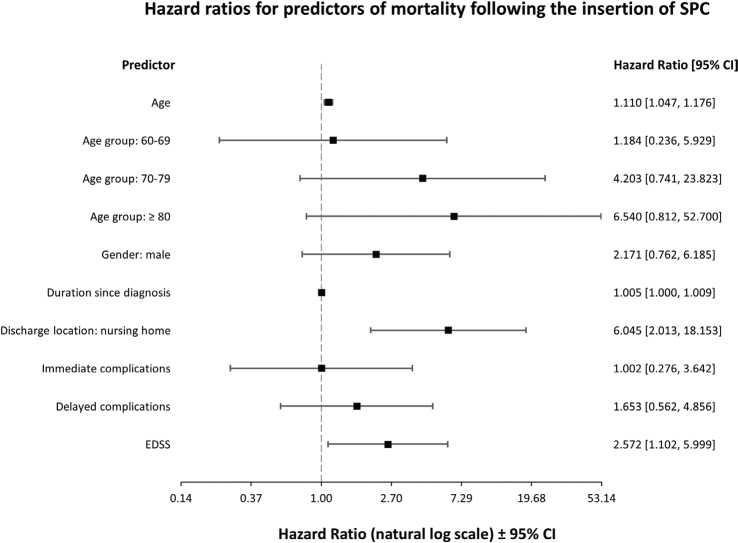
Forest plot detailing hazard ratios for individual predictors of post-suprapubic catheter (SPC) mortality in multiple sclerosis (MS) patients based on the univariable Cox regression model (*n* = 55). CI: confidence interval; EDSS: Expanded Disability Status Scale.

At the time of SPC insertion, 27% (*n* = 15) of patients were walking; 37 (67%) were wheelchair users. However, hazard ratios across all mobility levels were not significant (*p* > 0.05). Fig. 4 shows a forest plot detailing the hazard ratios and confidence intervals for individual predictors of mortality following SPC insertion in the MS patients.

Of those with MS, 49 (89%) were discharged to home after insertion of SPC. Six (11%) patients were discharged to nursing homes. There is a significant difference between the survival rate of patients with different discharge locations (log-rank [Mantel–Cox]: χ^2^=13.323, *p* < 0.001). Discharge to residential care predicted poorer survival (unadjusted HR = 6.05, *p* = 0.001; adjusted HR [age-adjusted] = 5.14, *p* = 0.004).

The most prevalent primary cause of death (1a) was pneumonia/Covid-19 infection (*n* = 6, 40%), followed by multiple sclerosis (*n* = 2), and 1 each from Proteus septicaemia following urinary tract infection, oesophageal cancer, and pancreatitis. The cause of death could not be ascertained from records in 4 patients. One death attributed to urinary infection occurred at 630 days after the insertion of SPC.

## DISCUSSION

To our knowledge, this is the first study to examine survival following radiologically guided SPC insertion in people with MS. Radiological insertion of SPC is increasingly used as a minimally invasive alternative to surgical placement, particularly in patients with significant comorbidity or anaesthetic risk. The procedure was associated with acceptable rates of immediate and delayed complications, with approximately two-thirds of patients alive 5 years after insertion. Older age, higher EDSS scores, and discharge to residential care were associated with poorer survival.

Radiological insertion of SPC is a minimally invasive, image-guided procedure performed under local anaesthesia in an outpatient setting. Real-time ultrasound or fluoroscopic guidance ensures precise needle placement into the urinary bladder through the lower abdominal wall, significantly reducing the risk of vascular and bowel injury compared with conventional surgical insertion performed under general anaesthesia in an operating theatre setting. The radiological approach is therefore associated with improved procedural success rates and avoids the risks associated with general anaesthesia, which is particularly relevant in patients with advanced MS who may have significant cardiorespiratory comorbidities. However, radiological SPC insertion is not suitable for all patients. Those who require general anaesthesia due to inability to tolerate the procedure under local anaesthesia, significant procedural anxiety, or previous lower abdominal surgery would be more appropriately managed with conventional surgical insertion under urological supervision.

The indications for radiological SPC insertion in our MS cohort were largely multifactorial, highlighting the complex interplay of functional decline and urological symptom burden in advanced stages of MS. Three in every 5 patients (*n* = 31/55) who underwent insertion of SPC experienced a deterioration in their ability to perform IC over time. This is due to deterioration in motor function, resulting in weakness of hands, impaired hand dexterity, loss of trunk control, reduced mobility, and challenges with transfers. These findings align with previous studies suggesting that as disability advances, the feasibility and sustainability of IC decrease, necessitating consideration of more permanent catheterization strategies such as SPC (22). A proportion of patients (35%) were on an indwelling urethral catheter, with most undergoing elective conversion to SPC. This reflects a common clinical pathway in which SPC is considered a more sustainable long-term solution compared with IUC, offering potential benefits such as reduced urethral trauma, improved patient comfort, and potentially lower rates of UTIs (11, 23).

The rate of immediate complications following SPC was similar to or lower than the figures previously reported (24, 25). Hobbs et al. (26) reported a 59% complication rate across 1,000 elective SPC procedures over 17 years. Our lower incidence may be partly attributable to the use of radiological guidance for SPC insertion. Radiologically guided SPCs are performed under local anaesthetic and/or sedation, enabling rapid recovery, and may be more appropriate for patients with significant comorbidities who are at high anaesthetic risk (27).

Radiologically inserted SPCs are performed without a cystoscopy, so any pre-existing debris within the urinary bladder is not washed out. It could therefore be hypothesized that early blockage could pose a greater risk in a radiologically inserted SPC than in a surgically inserted SPC where cystoscopy is used. In our cohort, 2 patients with MS experienced catheter blockage in the immediate period after SPC. None experienced major immediate complications such as bowel injury or death. Ahluwalia et al. (24) reported a 30-day mortality rate of 1.8% following insertion of SPC. In our study, there were no deaths in the first 30 days after the procedure.

Patients with prior catheter use experienced a statistically significantly higher rate of immediate complications compared with those without prior catheter use (24% vs 0%, *p* = 0.024). This may reflect a greater burden of pre-existing urinary tract pathology, including recurrent infections and urinary bladder changes associated with prolonged catheter use, which could predispose to procedural complications. However, given the small sample size and the exploratory nature of this comparison, this finding warrants further investigation in larger prospective studies.

Delayed complications were infrequent in our cohort, with low rates of bypassing, catheter-related infections, and urological admissions. These results support the long-term safety of SPC in MS. We also found that most individuals with MS who required SPC had high EDSS scores, indicative of advanced MS. High EDSS scores were not associated with higher risk of immediate or delayed complications. This suggests that advanced disability should not deter clinicians from offering SPC when clinically appropriate.

The development of urinary bladder or renal calculi is a recognized complication in individuals with long-term indwelling catheters, with several studies reporting incidence rates ranging from 22% to 65% (12, 28). Stone formation is commonly associated with colonization by urease-producing organisms such as *Proteus mirabilis*, which contributes to crystalline biofilm development. These bacteria hydrolyse urea to ammonia, increasing urinary pH and promoting the precipitation of calcium and magnesium phosphates, ultimately leading to catheter encrustation and stone formation (29). Only 2 patients in our cohort had cystolithiasis. The lower incidence observed in our cohort may reflect the shorter follow-up period, which may not have been sufficient for this complication to manifest.

More than 70% of individuals with MS survived over 5 years following the insertion of SPC. This high survival rate may be attributed to the younger age at SPC insertion among MS patients. Additionally, it could be due to the early involvement of urological care in MS. The increased awareness of neuro-urological complications among MS healthcare teams is likely to result in the early referral for SPC insertion. When considering SPC insertion, it is crucial to consider the long-term survival prospects of individuals with MS.

Age emerged as a significant predictor influencing survival in this study, likely reflecting the expected progression of disability and the increasing burden of comorbidities with advancing age. Sex did not impact survival, which is a similar observation to that found in our previous study on survival after gastrostomy placement for patients with MS (30).

Poor mobility status also did not significantly affect survival, suggesting that reduced mobility should not be a barrier to SPC consideration in this group. Our study also demonstrated that higher EDSS scores were significantly associated with reduced survival following the insertion of SPC in MS patients. Higher EDSS scores often reflect greater frailty and reduced physiological reserve, making these individuals more prone to adverse urological outcomes. Moreover, previous evidence from a retrospective observational study conducted in Switzerland has shown that higher EDSS scores are associated with an increased likelihood of presenting with at least 1 urodynamic risk factor for upper urinary tract (UUT) deterioration, further supporting this link (31). Need for residential and nursing care predicted poor survival in people with MS following the insertion of SPC. This is in line with the 2011 Census for England and Wales, which revealed that care home residents had a lower life expectancy across all age groups in both males and females (32). Discharge to institutional care may reflect pre-existing frailty and late-stage disease, and therefore likely represents a marker of poor prognosis rather than a causal determinant.

The median duration from MS diagnosis to SPC insertion of 17.5 years likely reflects the stepwise nature of NLUTD management, with SPC considered only after other strategies have been exhausted. Whether earlier SPC insertion could improve outcomes warrants further investigation in prospective studies. Duration since MS diagnosis also showed a statistically significant association with mortality following SPC insertion. This relationship remained significant when adjusted for age-group categories, indicating a 1% increase in the risk of mortality for each additional year since MS diagnosis, suggesting a small but potentially cumulative effect on survival over time. However, the association lost statistical significance when adjusted for other key covariates, including age (as a continuous variable), EDSS score, and discharge to residential care (used as a proxy for frailty). These findings suggest that the prognostic value of disease duration may be confounded by interrelated factors such as age, disability status, and care needs, all of which are closely linked to the overall course of MS. Given the small effect size and loss of significance in multivariable models, duration of MS diagnosis should be interpreted cautiously as an independent predictor of mortality in this context. These results also emphasize the complexity of modelling survival in a small cohort with overlapping clinical characteristics and highlight the need for larger studies to better explain the independent prognostic role of disease duration following SPC insertion.

Respiratory infection was the most common cause of death in this cohort, aligning with previous studies identifying respiratory complications as a leading cause of MS-related mortality (33, 34). The timeframe of this study overlapped with the COVID-19 pandemic, which likely influenced mortality rates during the follow-up period. Two deaths were related to COVID-19 infection throughout this study (2/15 deaths; 13%). The risk of death involving COVID-19 was 1.4–1.6 times greater for people with significant disabilities in England (35).

### Limitations

This study has several limitations. It is a small, single-centre retrospective analysis and therefore relies on information available in hospital records rather than prospectively collected data. Some potentially relevant variables were not captured. Quality-of-life outcomes and medical comorbidities, which may influence survival, were not routinely recorded and could not be evaluated. We have no data on patients who were offered SPC but declined, or on those managed with IC or indwelling urethral catheterization during the same period. This selection bias means the cohort may not be representative of the broader MS population requiring urinary bladder management. Therefore, conclusions regarding the relative benefits of SPC over alternative urinary bladder management strategies cannot be drawn. Some 145 non-MS patients underwent radiologically guided SPC insertion at our centre during the same period; future studies could use this group as a comparator to better contextualize these findings.

Additionally, as data were derived solely from hospital records at a single tertiary centre, complications managed in primary care or by community nursing teams may not have been fully captured, potentially leading to under-reporting of events such as catheter-associated urinary tract infections or catheter expulsions. Urodynamic data were not available for all patients; 1 patient with overactive urinary bladder symptoms could not have NDO formally confirmed. It was also not possible to confirm from available records whether patients received instructions regarding urinary bladder irrigation as part of their catheter care. Our centre has a dedicated neuro-urology service delivered by a urology consultant and MS nurse specialists for people with MS. This specialized service may have facilitated better patient selection and closer monitoring, potentially leading to lower complication rates and improved outcomes. Consequently, these results may not be generalizable to centres without similar multidisciplinary expertise.

Retrospective ascertainment of cause of death is subject to inaccuracy. Death certificates were used as the primary source; however, the cause of death could not be ascertained in 4 of 15 patients (27%), which limits the completeness of mortality data. The small sample size and limited number of deaths restricted the complexity of survival modelling. Regression analyses were therefore deliberately limited to parsimonious models to minimize the risk of overfitting. Small subgroup sizes also resulted in wide confidence intervals for some predictors, reducing statistical power and potentially obscuring true associations. The survival analyses should therefore be interpreted as exploratory.

### Conclusion

People with MS who underwent SPC insertion under radiological guidance had favourable long-term survival and acceptable complication rates. Mortality appeared to be primarily related to underlying disease severity and frailty rather than complications of SPC itself. Older age, higher EDSS, and discharge to residential care predicted poorer survival following catheter insertion. Longer disease duration showed an association with mortality in univariable analysis, though this was attenuated after adjustment.

These findings may help clinicians and patients understand better the potential complications of suprapubic catheterization in advanced MS. Future studies should aim to validate these findings in larger prospective multicentre cohorts. Inclusion of quality-of-life outcomes would provide a more comprehensive understanding of the impact of SPC in MS. Further research stratifying outcomes by disease subtype and disability severity may help refine patient selection and optimal timing of catheter insertion. A national registry capturing urological interventions in MS could facilitate longitudinal analysis and guide best practices for management of NLUTD in MS.
